# Peripheral nerve injury mediated by JEV strain NX1889 infection and impairment of Schwann cells

**DOI:** 10.1371/journal.pntd.0013466

**Published:** 2025-08-26

**Authors:** Liping Yang, Yanping Yuan, Qikai Yin, Xiaocong Li, Xue Kang, Qianqian Cui, Guowei Wang, Huan Yang, Ruichen Wang, Tingting Yang, Fan Li, Shihong Fu, Huanyu Wang, Zhenhai Wang

**Affiliations:** 1 The First Clinical Medical School, Ningxia Medical University, Yinchuan, China; 2 Department of Critical Care Medicine, General Hospital of Ningxia Medical University, Yinchuan, China; 3 National Key Laboratory of Intelligent Tracking and Forecasting for Infectious Diseases, National Institute for Viral Disease Control and Prevention, Chinese Center for Disease Control and Prevention, Beijing, China; 4 Neurology Center, General Hospital of Ningxia Medical University, Yinchuan, China; 5 The First Affiliated Hospital of Chongqing Medical University, Chongqing, China; 6 NHC Key Laboratory of Diagnosis and Treatment on Brain Functional Diseases, Chongqing, China; 7 Emergency Center, General Hospital of Ningxia Medical University, Yinchuan, China; 8 Institute of Medical Sciences, General Hospital of Ningxia Medical University, Yinchuan, China; 9 Diagnosis and Treatment Engineering Technology Research Center of Nervous System Diseases of Ningxia, Yinchuan, China; Colorado State University, UNITED STATES OF AMERICA

## Abstract

Japanese encephalitis virus (JEV) is a mosquito-borne flavivirus primarily associated with central nervous system disorders. Recent studies have indicated that JEV infection can lead to Guillain-Barré syndrome (GBS), a peripheral nervous system (PNS) disorder; however, the underlying mechanisms remain unclear. In this study, we investigated the tropism of different genotypes of JEV strains for peripheral nerves and their potential impact on peripheral nerves. Four JEV strains—GI NX1889, GI GZ56, GIII P3, and GV XZ0934—representing three prevalent genotypes in China were selected for analysis. Compared with other JEV strains, NX1889 exhibited greater peripheral nerve tropism, leading to peripheral nerve dysfunction, severe pathological changes, and inflammatory responses. Further investigation revealed that Schwann cells (SCs) supported productive replication of GI JEV, but not GIII or GV strains. Although both GI strains infected SCs and caused proliferation inhibition and G1 phase arrest, only NX1889 induced apoptosis. At the symptomatic peak, NX1889-infected mice demonstrated significantly higher viral loads in sciatic nerves and dorsal root ganglia compared to peripheral non-neural tissues and organs. Sciatic nerves exhibited extensive infiltration of CD45 + inflammatory cells and upregulated pro-inflammatory mediators, and the viral load and CCL2 expression in sciatic nerves were positively correlated with limb motor deficits. These findings demonstrate that JEV strains exhibit distinct abilities to induce peripheral nerve injury (PNI), with the variation being a strain-specific characteristic of NX1889 rather than a genotype-specific feature of GI. The peripheral nerve is a vital site for JEV NX1889 replication, and the direct viral invasion of SCs leads to demyelinating neuropathy, with subsequent immune-inflammatory responses exacerbating this injury. This study broadens the spectrum of pathogens known to directly invade and damage peripheral nerves and provides new insights into the pathogenesis of JEV-associated PNI.

## Author summary

JEV is the causative agent of Japanese encephalitis, the most prevalent viral encephalitis in Asia. Emerging evidence indicates that JEV infection can also lead to PNI, highlighting its broader neurotropic potential. We aimed to investigate whether the increased incidence of JEV-associated PNI is attributed to the epidemiological shift of JEV GI as the dominant genotype or is specifically linked to the pathogenic characteristics of the NX1889 strain. Furthermore, we investigated the potential role of the interaction between JEV and SCs in the pathogenesis of JEV-associated PNI. We elucidated this by a comparative study regarding the PNS-specific impacts of JEVs with respect to viral susceptibility, virulence, and the antiviral inflammatory response using in vivo and in vitro model systems. Our findings revealed that, JEVs exhibited distinct capacities to induce PNI, with the observed variations primarily attributed to the strain-specific characteristics of NX1889 rather than the genotype-specific features of JEV GI. In addition to its well-established central nervous system (CNS) tropism, the PNS was also a vital site for JEV NX1889 replication, and the direct invasion of SCs by JEV NX1889 contributes to peripheral demyelination pathology.

## 1 Introduction

JEV causes Japanese encephalitis (JE), the most common vector-borne viral disease in Asia and the Western Pacific; it has the potential for global spread. Approximately 69,000 JE cases are reported annually, and the average mortality rate is 18% over the past 30 years [1]. JEV, a flavivirus in the *Flaviviridae* family, is classified into five genotypes (GI–GV) based on genomic sequence. Currently, only the GI, GIII, and GV genotypes are prevalent in China. Over the past two decades, JEV GI has gradually replaced GIII as the dominant genotype in typical Asian epidemic regions [2]. In 2018, a JEV GIb strain, NX1889, was isolated from a patient’s cerebrospinal fluid (CSF) during an outbreak of JEV-associated GBS in Ningxia [3]. Strain NX1889 and other JEV strains isolated from local mosquito during the outbreak formed a distinct branch representing a new phylogenetic group in the evolution of JEV GIb [3]. Phylogenetic analysis of GI JEV isolates from 2016 to 2021 indicated greater activity in northern China than in southern China; most GIb strains, especially in northern China, belonged to an emerging subclade [4].

JEV is primarily associated with central nervous system (CNS) disorders. However, recent reports of GBS associated with JEV infection highlight the changing clinical characteristics of JEV-induced disease. In the past two decades, JEV-associated GBS mainly was reported in sporadic cases [5–7]. In 2018, an outbreak of JEV-associated GBS occurred [8]. A retrospective study of JE patients from 2016 to 2020 in China showed that 14% of patients with JEV infection exhibited PNI. These patients were mainly adults located in northwestern China presenting with flaccid paralysis and severe respiratory muscle paralysis; they had a higher risk of respiratory failure and worse prognosis relative to patients with JE alone [9].

In previous studies, we demonstrated that injection of JEV GI NX1889 in mice could induce PNI [10,11]. By integrating the spatiotemporal phylogenetics of JEV GI with the temporal and geographic distribution of JEV-associated PNI cases, it was observed that both the endemic regions of GI JEV and the occurrence of JEV-associated PNI cases have been predominantly concentrated in northern China in recent years [4,9]. This spatiotemporal coincidence, combined with the finding that JEV GI NX1889 can induce PNI, prompted us to investigate whether the increased incidence of JEV-associated PNI is attributed to the epidemiological shift of JEV GI as the dominant genotype or is specifically linked to the pathogenic characteristics of the NX1889 strain. To address this question, we selected four JEV strains representing three prevalent genotypes in China, including two GI strains (NX1889 and GZ56), for a comprehensive comparative study on their viral susceptibility, virulence, and antiviral inflammatory responses targeting the peripheral nerves, thereby elucidating their distinct impacts on the PNS.

Most patients with GBS exhibit a history of preceding infection, but only a limited number of pathogens have been shown to cause neurological damage by direct invasion in GBS. It is widely believed that the pathogenesis of GBS is mediated by post-infectious immune-inflammatory responses [12]. Nevertheless, a study proved that Zika virus (ZIKV), which has been associated with GBS outbreaks [13], can directly infect peripheral neurons and induce cell death [14]. Our previous studies have indicated that immunoinflammatory responses are involved in the pathogenesis of JEV-associated PNI [15,16]. Given that both JEV and ZIKV are both neurotropic flaviviruses, we hypothesized that JEV-associated GBS might also result from direct viral invasion. Since JEV-associated PNI is primarily characterized by demyelination [10,11], and SCs are the myelinating glial cells of the PNS, we focused on SCs as the primary target of our investigation. We aimed to determine whether JEV strains can replicate in SCs and whether such replication directly contributes to PNI using both in vivo and in vitro model systems. Specifically, we explored the interactions between JEVs and SCs, and analyzed the relationships among viral replication, inflammatory responses, and limb motor deficits to provide mechanistic insights into JEV-associated PNI.

## 2 Materials and methods

### 2.1 Ethics statement

This study was approved by the Ethics Review Committee of the National Institute for Viral Disease Control and Prevention of the Chinese Center for Disease Control and Prevention (China CDC, approval numbers bdbs0120240126003 and 20220420043) and carried out in strict accordance with the requirements of the ethics committee.

### 2.2 Cells and viruses

Baby hamster kidney (BHK)-21 cells were cultured in minimum essential medium (MEM, Gibco), supplemented with 10% fetal bovine serum (FBS, Sigma-Aldrich), 1% penicillin, and streptomycin (100 U/mL). Rat Schwann cells (RSC96 cells) were grown in dulbecco’s modified eagle medium (DMEM, Gibco), supplemented with 10% FBS, 1% penicillin, and streptomycin (100 U/mL). BHK-21 cells were kept in our laboratory and RSC96 cells were purchased from ATCC.

All JEV strains were previously isolated and maintained in the pathogenic microorganism strain preservation center of the China CDC (S1 Table). The NX1889 strain was isolated from the cerebrospinal fluid (CSF) of an adult patient with JE combined with GBS in 2018. It is currently the only JEV strain isolated from a JE patient with GBS and is associated with a GBS outbreak. The GZ56 strain was isolated from the CSF of an infant patient with JE in 2008. The P3 strain was isolated from a fatal JE case in 1950. It is one of the earliest JEV strains isolated and preserved in China and serves as the classic strain for inactivated JE vaccine production. The XZ0934 strain, isolated in 2009 from mosquito specimens, is the only JEV GV strain in China. All JEV strains have undergone plaque purification to ensure genetic homogeneity and experimental reliability (S1 Fig).

### 2.3 Experimental mice

Male C57BL/6N mice aged 4–5 weeks were purchased from Vital River and housed in the specific pathogen-free environment in the Animal Center of the China CDC. In the first cohort of experiments, 60 mice were randomly divided into five groups and intraperitoneally inoculated with MEM (mock control) or 1 × 10^5^ PFU JEV NX1889, GZ56, P3, and XZ0934. Half of the mice in each group were euthanized at 10–12 days post-infection (dpi) for tissue collection and subsequent analysis, while the remaining mice were monitored for 14 days to evaluate the general conditions (coat condition, weight, and active state), limb paralysis, and survival status. In the second cohort of experiments, 30 mice intraperitoneally inoculated with JEV NX1889 and 3 control mice were assessed and sampled on 8–12 dpi. A 20% reduction in initial body weight was utilized as the termination criteria, with these mice being euthanized and classified as death.

### 2.4 Behavioral motor function

The viral paralysis scale (VPS) was used to assess paralysis of the tail and limbs. Severity was assessed and graded from 0 to 6 based on the S2 Table. A score of 0 is considered normal and a score of 6 is considered complete paralysis or death. Neuromuscular strength and coordination were evaluated using a hanging wire test (HWT). A 50 cm high metal net was placed above the table. Remaining on the metal net for 180 s was considered normal. The falling time was recorded when the mouse fell during this period.

### 2.5 Electromyography

After anesthetization, bilateral sciatic nerves were carefully stripped from the muscle. A stimulating needle electrode of the proximal site was positioned near the sciatic nerve root and a second stimulating of distal site was placed at the sciatic notch, while a recording needle electrode was inserted into the ventral gastrocnemius muscle. The compound muscle action potential was recorded by electromyography (NDI-094 Shanghai Haishen).

### 2.6 Histopathology

Brain, sciatic nerve and DRG tissues were fixed in 4% paraformaldehyde, embedded in paraffin wax, and sectioned. The sections were stained using Hematoxylin and Eosin (HE) or incubated with the anti-CD45 antibody (1:300, Servicebio, GB113886) followed by the corresponding secondary antibody. Images were captured using a fluorescence microscope (Mshot, MF53-N).

### 2.7 Immunofluorescence

RSC96 cells were harvested, dropped onto slides and fixed in acetone. For tissues, after deparaffinization, rehydration, antigen retrieval, the sciatic nerve and DRG sections were blocked with 3% BSA. The cell slides or tissue sections were incubated with the primary antibodies as follows: anti-JEV NS1 antibody 1:200 (Genetex, GTX633820), anti-myelin basic protein (MBP) antibody 1:50 (Cell Signaling Technology, 78896s), anti-cleaved caspase-3 (cl-Casp3) antibody 1:400 (Cell Signaling Technology, 9964s), anti-s100β antibody 1:2000 (Servicebio, GB11359), and anti-β3-Tubulin (clone Tuj1) antibody 1:400 (Cell Signaling Technology, 5568s). After washes, the cell slides or sections were incubated with the secondary antibodies as follows: CY3 goat anti-mouse IgG 1:300 (Servicebio, GB21301), Alexa Fluor 488 goat anti-rabbit IgG 1:400 (Servicebio, GB25303), FITC goat anti-mouse IgG 1:500 (Absin, abs20003), FITC goat anti-rabbit IgG 1:200 (Absin, abs20004), and Alexa Fluor 594 goat anti-rabbit IgG 1:500 (Jackson, 163359). The cell slides or sections were stained with DAPI, and finally captured under a fluorescence microscope (Nikon eclipse C1, Mshot MF53-N), or a confocal scanning laser microscope (Nikon, Ti-E + A1 MP). Images were analyzed by Image J software to measure the fluorescence levels.

### 2.8 Plaque‑forming assay

Virus-containing samples were diluted in MEM and then incubated with BHK-21 cells in 6-well plates at 37°C with 5% CO_2_ for 60 min. The wells were washed and received 3 mL of 1% methylcellulose maintenance medium containing 2% FBS, 1% penicillin, and streptomycin (100 U/mL). After 4 days of incubation, cells were fixed with 2% crystal violet for 30 min and rinsed with tap water.

### 2.9 RNA extraction and qRT-PCR

Viral RNA of RSC96 cell or serum was extracted using the nucleic acid extraction kit (Tianlong Biotechnology, Jiangsu, China). The total RNA of RSC96 cell was extracted by the total RNA kit I (Omega Bio-tek, R6834-02). The tissues and organs were pulverized repeatedly in liquid nitrogen and the total RNA was extracted using Trizol reagent (Invitrogen, 59203). Viral RNA was tested by One Step qRT-PCR probe kit (Vazyme Biotech, Q225). The mRNA of inflammatory cytokines was quantified using One Step qRT-PCR SYBR Green kit (Vazyme Biotech, Q211). The primers for JEV covered all the JEV genotypes as published [17]. The primer sequences for cytokines are provided in S3 Table.

### 2.10 Cell proliferation test

RSC 96 cells were inoculated into 96-well plates at a density of 1 × 10^4^ cells/well. After 24 h, RSC96 cells were infected with JEV strains for 60 min. Subsequently, the virus-containing supernatants were removed, and cells were further incubated with DMEM complete medium. The cell proliferation was tested by Cell Counting Kit-8 (Dojindo, CK04) according to the instruction. Cell viability (%) = (OD value of experimental well - OD value of blank well)/ (OD value of control well - OD value of blank well) × 100%. Experimental well: contains infected RSC96 cells, culture medium, and CCK8; Control well: contains uninfected RSC96 cells, culture medium, and CCK8; Blank well: contains only CCK8 and culture medium, without cells.

### 2.11 Cell cycle detection

RSC96 cells were inoculated into 6-well plates at a density of 3 × 10^5^ cells/well. After 24 h, RSC96 cells were infected with JEV strains for 60 min. After removing supernatants, RSC96 cells were added with DMEM complete medium and incubated for 24 h, 30 h, or 36 h separately. The samples were harvested and fixed with 70% ethanol solution overnight. Then the samples were incubated with 50 μL of RNase A (100 μg/mL) (Thermo, 12091021) at 37°C for 30 min, and with 450 μL of PI solution (50 μg/mL, containing 0.1% Triton X-100) (Sigma, P4170) for 30 min. The prepared samples were detected by flow cytometry (Cytek NL-CLC3000, USA).

### 2.12 Cell apoptosis detection

RSC96 cells were seeded into 6-well plates at a density of 2.5 × 10^5^ cells/well. After 24 h, cells were infected with JEV strains for 60 min. After removing supernatants, RSC96 cells were added with DMEM containing 2% FBS, 1% penicillin, and streptomycin (100 U/mL) and incubated for 60 h, 72 h, or 96 h separately. Apoptosis detection was performed according to the instructions of Annexin V-FITC/PI Apoptosis Detection Kit (4A Biotech, FXP018Pro). The cell samples were promptly detected by flow cytometry (Cytek NL-CLC3000, USA).

### 2.13 Statistical analysis

Statistical analysis was performed by GraphPad Prism, and all the data were expressed as mean ± standard error of the mean (SEM). Unpaired two-tailed Student’s *t*-test or one-way analysis of variance (ANOVA) was used to compare two or multiple groups for one factor. For comparisons of multiple factors, two-way ANOVA was performed. VPS scores, JEV RNA loads, and cytokines were evaluated by Pearson’s correlation. P-values of < 0.05 were considered statistically significant. (*P < 0.05, ** *P* < 0.01, ***P < 0.001, and ****P < 0.0001).

## 3 Results

### 3.1 Strain NX1889 induces motor deficits and functional impairments in peripheral nerves

**Fig 1:**
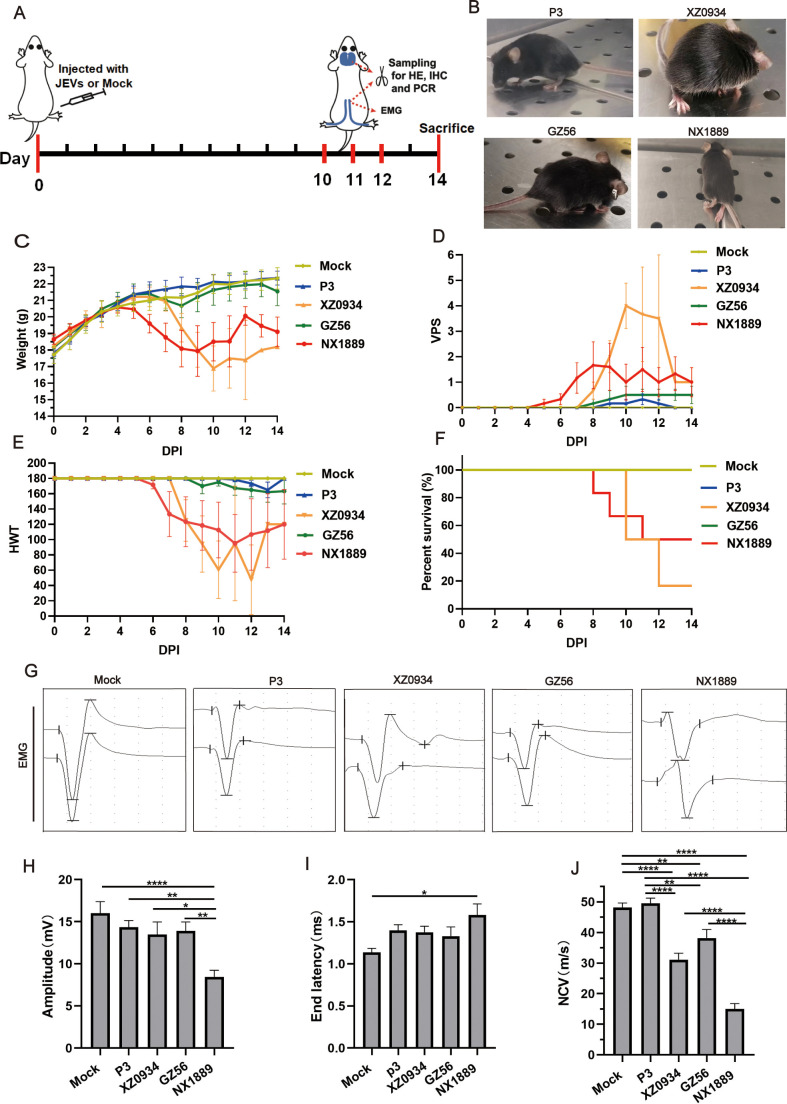
General conditions and motor function assessment of peripheral nerves in JEVs-infected mice. (A) Experimental timeline of murine model. Mice were intraperitoneally inoculated with MEM (mock control) or 1 × 10^5^ PFU JEV NX1889, GZ56, P3, and XZ0934 strains. In each group, half of the mice were euthanized at 10-12 dpi to perform sciatic nerve electromyography, HE staining, immunohistochemistry, and qRT-PCR. The remaining cohort was monitored daily for general conditions, neurological function and survival status over a 14-day observation period. (B) Representational pictures of JEVs-infected mice during peak illness. (C-F) Daily monitoring of the body weight change, VPS scores, HWT scores, and survival curves of JEVs-infected mice or mock control over a 14-day period. VPS and HWT scores were used to assess the limb strength of mice; **n* *= 6. (G) Representative electrophysiological recordings of the sciatic nerve compound muscle action potentials (CMAPs) of JEVs-infected mice during peak illness. (H-J) Statistical analysis of the bilateral sciatic nerve CMAP in amplitude, end latency and NCV.

Mice were intraperitoneally inoculated with MEM (mock control) or 1 × 10^5^ PFU JEV strains, and monitored daily for general conditions, including coat condition, body weight, and activity levels, as well as limb paralysis and survival status (Fig 1A). The VPS and HWT were employed to quantitatively assess the severity of limb paralysis. Mice infected with JEV strains began to exhibit symptoms on 5 days post-infection (dpi), with the most severe symptoms observed between 8 and 12 dpi. Among the four infection groups, the GV XZ0934 and GI NX1889 groups exhibited more pronounced general deterioration, more severe limb paralysis (as evidenced by worse VPS and HWT scores), and reduced survival rates, whereas the GIII P3 and GI GZ0656 groups experienced relatively milder symptoms (Fig 1B-1F). The XZ0934 group primarily manifested severe encephalitis and systemic wasting symptoms, whereas the NX1889 group, even in the absence of systemic wasting symptoms, exhibited unilateral or bilateral limb motor impairment in some mice, including complete limb paralysis in severe cases. Due to overlapping symptoms, it is often challenging to distinguish between CNS and PNS lesions in mice solely based on behavioral observations and limb strength. Therefore, we performed electromyography to evaluate motor function in sciatic nerves (Fig 1G and S4 Table). Among the four JEV-infected groups, only NX1889-infected mice displayed a significant reduction in amplitude (Fig 1H) and prolonged end latency (Fig 1I). All four JEV strains caused decreased nerve conduction velocity (NCV); notably, the NCV in the NX1889 group was reduced by more than half compared with the mock group (14.98 ± 1.76 m/s versus 48.1 ± 1.50 m/s, *P* < 0.0001) (Fig 1J). In summary, GIII and GV strains caused mild demyelination in the sciatic nerve without axonal injury; regarding the two GI strains, only NX1889 caused severe demyelination accompanied by axonal injury.

### 3.2 Strain NX1889 causes severe pathological changes in the PNS

**Fig 2:**
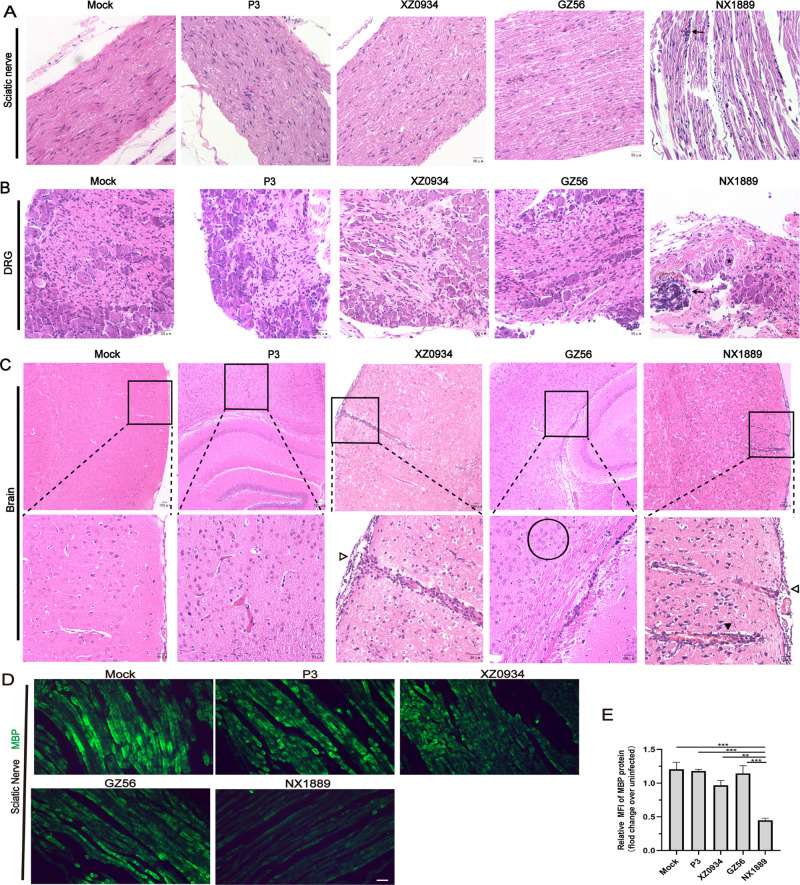
Neuropathological alterations in the sciatic nerve, DRG, and brain of JEVs-infected mice during peak illness. (A-C) Representative images of HE staining of the (A) sciatic nerve, (B) DRG and (C) brain. ↓ infiltration and aggregation of inflammatory cells,▼perivascular cuffing accompanied by congestion, ∇lymphocytic infiltration in the meninges, ★neuronal damage, 〇gliosis. (D, E) Representative images and statistical analysis of MBP in the sciatic nerve. *n* = 3. Scale bar for (A, B) 50 μm, (C) 100 μm (up) and 50 μm (down), and (D) 20 μm.

During peak illness, no typical pathological damage induced by JEV GIII or GV strains was observed in the sciatic nerve (Fig 2A) or dorsal root ganglion (DRG) (Fig 2B). Between the two GI groups, only the NX1889 group exhibited severe pathological damage in the sciatic nerve and DRG, characterized by vascular congestion, hemorrhage, edema, loose and irregular arrangements of nerve fibers, neuronal damage, and infiltration and aggregation of inflammatory cells. Pathological changes were observed in the brains of all four infected groups. The GV XZ0934 and GI NX1889 groups displayed extensive neuronal degeneration, necrosis and vacuolization, accompanied by obvious vascular congestion, perivascular cuffing, and widespread lymphocytic infiltration in the meninges and brain parenchyma. The GIII P3 and GI GZ56 groups exhibited partial neuronal degeneration and necrosis, gliosis, dilation of the perivascular spaces, and edema (Fig 2C). At the molecular level, compared to the mock group and the GI GZ56, GIII P3, and GV XZ0934 group, the GI NX1889 group showed a significant reduction in myelin basic protein (MBP) levels in the sciatic nerve (Fig 2D and 2E), indicating severe myelin destruction induced by NX1889 infection.

### 3.3 Strain NX1889 triggers robust inflammatory responses in the PNS

**Fig 3:**
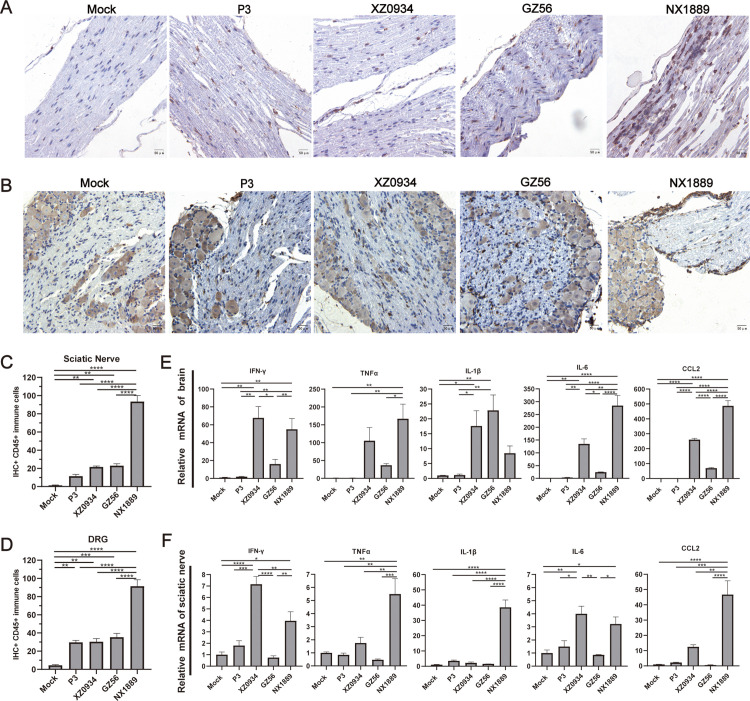
Assessment of neuroinflammatory responses of JEVs-infected mice during peak illness. (A-D) Representative images of immunohistochemical staining for CD45 and quantification of CD45 + immune cells in the (A, C) sciatic nerve and (B, D) DRG. The quantitative analysis of CD45 staining was performed by counting CD45-positive cells. CD45-positive cells were stained brown. DRG neurons, which are larger in volume than CD45-positive cells, exhibited nonspecific staining, with their cytoplasm stained in a light brown color. *n* = 5. (E, F) The mRNA expression of pro-inflammatory factors in the brain and sciatic nerve. *n* = 3. Scale bar is 50 μm.

During peak illness, the GI NX1889 group showed substantial infiltration and aggregation of CD45 + cells in both the sciatic nerve and DRG, whereas the GIII P3, GV XZ0934, and GI GZ56 group showed minimal CD45 + cells infiltration (Fig 3A and 3B). Statistical analysis revealed that the number of CD45 + cells in the NX1889 group was significantly higher compared to the mock group and the GI GZ56, GIII P3, and GV XZ0934 group (Fig 3C and 3D). We further examined pro-inflammatory factors in both the central and peripheral nervous systems during the symptomatic peak. In the brain tissues, the GV XZ0934 and GI NX1889 groups exhibited higher overall inflammatory responses compared to the GIII P3 and GI GZ56 groups (Fig 3E). In the sciatic nerves, the NX1889 group demonstrated the most robust inflammatory responses (Fig 3F). In the sciatic nerves, the NX1889 group showed marked elevations in IFN-γ, TNF-α, IL-6, IL-1β, and CCL2 levels compared to the mock control, with particularly pronounced increases in IL-1β and CCL2 (Fig 3F).

### 3.4 The PNS is a vital site for the replication of strain NX1889

**Fig 4:**
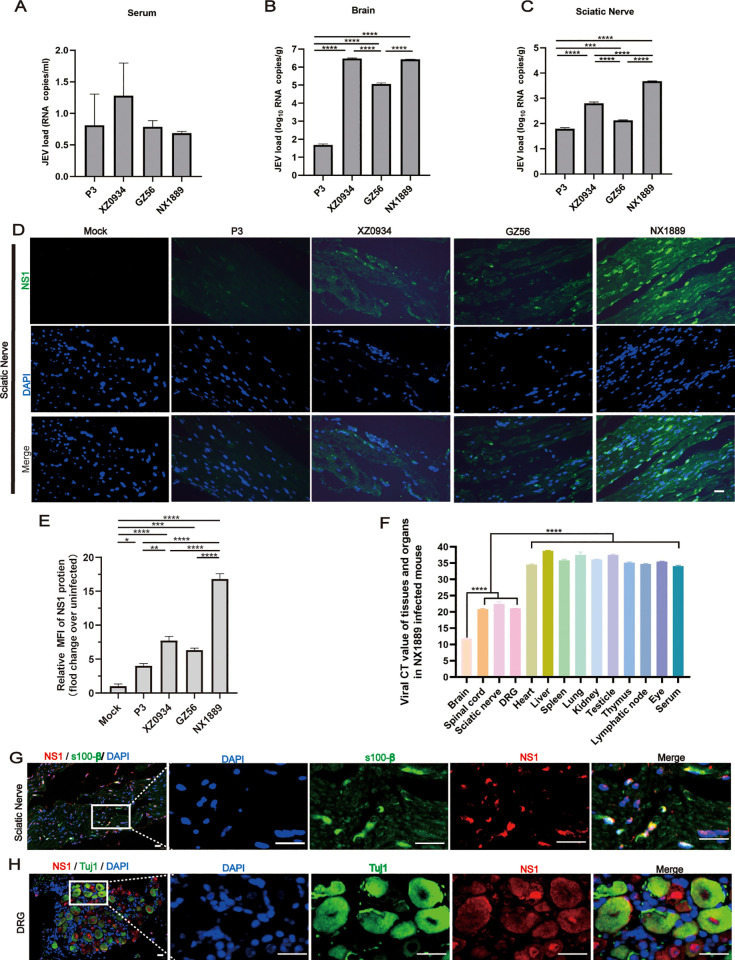
Viral loads of tissues and organs in JEVs-infected mice. (A-C) JEV RNA load of the (A) serum, (B) brain, and (C) sciatic nerve in JEVs-infected mice during peak illness. JEV RNA load was detected by qRT-PCR. (D, E) Representative images and statistical analysis of JEV-NS1 protein in the sciatic nerve of JEVs-infected mice during peak illness. (F) CT value of tissues and organs in JEV NX1889-infected mice, including tissues of the CNS, i.e., brains and spinal cords, tissues of the PNS, i.e. DRGs and sciatic nerves, and peripheral organs or tissues. (G) Visualization of SCs, co-stained with JEV NS1 protein in the sciatic nerve of NX1889-infected mice. (H) Visualization of neurons, co-stained with JEV NS1 protein in the DRG of NX1889-infected mice. SCs labeled by s100β and DRG neurons labeled by Tuj1. *n* = 3. Scale bar is 20 μm.

During peak illness, JEV RNA levels were markedly low in serum samples from JEVs-infected mice (Fig 4A). JEV RNA levels were similar between the GI NX1889 and GV XZ0934 group in the brain tissues; the viral levels of brain in these two groups were significantly higher than in the GI GZ56 and GIII P3 groups (Fig 4B). In the sciatic nerves, the GI NX1889 group displayed markedly elevated JEV RNA levels compared to the GI GZ56, GIII P3, and GV XZ0934 group (Fig 4C). Immunofluorescence analysis revealed the expression level of JEV NS1 protein was significantly higher in the NX1889 group compared to the GI GZ56, GIII P3, and GV XZ0934 groups, consistent with the qRT-PCR results (Fig 4D and 4E). Given the observed susceptibility of JEV NX1889 to peripheral nerves, we systematically quantified viral loads in both central and peripheral tissues and organs of NX1889-infected mice during the peak symptomatic period. The highest viral load was detected in brain tissue, followed by the spinal cord, DRG, and sciatic nerve; conversely, non-neural tissues and organs exhibited negligible levels of JEV RNA (Fig 4F), suggesting that the PNS was a potential site of JEV NX1889 replication and a tissue-specific target for infection. To visualize viral distribution within the PNS, multiplex immunofluorescence staining was performed. The results demonstrated co-localization of S100β and JEV NS1 in the sciatic nerves, as well as Tuj1 and NS1 in the DRGs, confirming NX1889 could infect SCs in the sciatic nerve (Fig 4G) and neurons in the DRG (Fig 4H).

### 3.5 SCs exhibit susceptibility to infection with GI JEV

**Fig 5:**
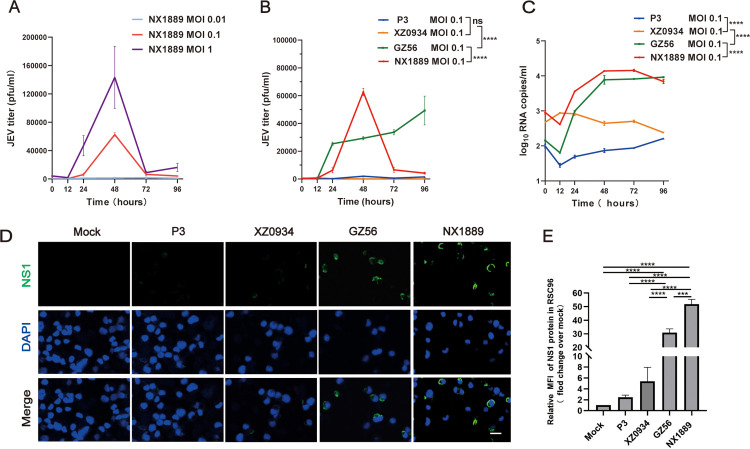
Replication of JEVs in RSC96 cells. (A) Growth curves of strain NX1889 in RSC96 cells. RSC96 cells were infected with JEV NX1889 at MOIs of 0.01, 0.1, and 1. Viral titers were quantified by plaque assay on BHK-21 cells at 12, 24, 48, 72, and 96 hpi. (B) Comparison of growth curves of four JEV strains (MOI = 0.1) in RSC96 cells assessed by plaque assay. (C) Comparison of growth curves of four JEV strains (MOI = 0.1) in RSC96 cells detected by qRT-PCR. (D, E) Representative immunofluorescent staining images and statistical analysis of JEV NS1 protein expression in RSC96 cells at 48 hpi (MOI = 1). *n* = 3. Scale bar, 20 μm.

To further clarify the relationship between viral infection and peripheral nerve injury, we conducted in vitro model experiments using SCs. Initially, we investigated whether JEV NX1889 could replicate in SCs. RSC96 cells were infected with NX1889 at varying multiplicities of infection (MOI). When the MOI was ≥ 0.1, a significant increase in viral titer was observed. The viral titer began to rise at 12 hours post-infection (hpi) and peaked at 48 hpi, reaching (1.43 ± 0.44) × 10^5^ pfu/mL (Fig 5A). Subsequently, four JEVs representing three genotypes were incubated with RSC96 cells at an MOI of 0.1. The results of plaque assays showed that RSC96 cells were more susceptible to infection with GI N1X889 and GZ56 than with GIII P3 or GV XZ0934 (*P* < 0.0001) (Fig 5B). Intriguingly, the growth trend significantly differed between the two GI strains (*P* < 0.0001). In the NX1889 group, the viral titer exhibited a rapid increased post-infection, followed by a sharp decline at 48 hpi. In contrast, the GZ56 group showed a continuous and gradual rise in viral titer within 96 h (Fig 5B). We next performed qRT-PCR to quantify the viral load in JEV-infected SCs. The results revealed that viral RNA levels began to increase at 12 hpi, and RSC96 cells were more susceptible to infection with GI than with GIII or GV strains (Fig 5C), consistent with the results obtained from plaque assays. Immunofluorescence staining of NS1 protein in JEV-infected RSC96 cells at 48 hpi revealed that, a significant amount of JEV NS1 protein was observed in GI NX1889 and GZ56, predominantly localized in perinuclear region of the cells (Fig 5D and 5E).

### 3.6 GI JEV infection inhibits SCs proliferation and induces G1 phase arrest

**Fig 6:**
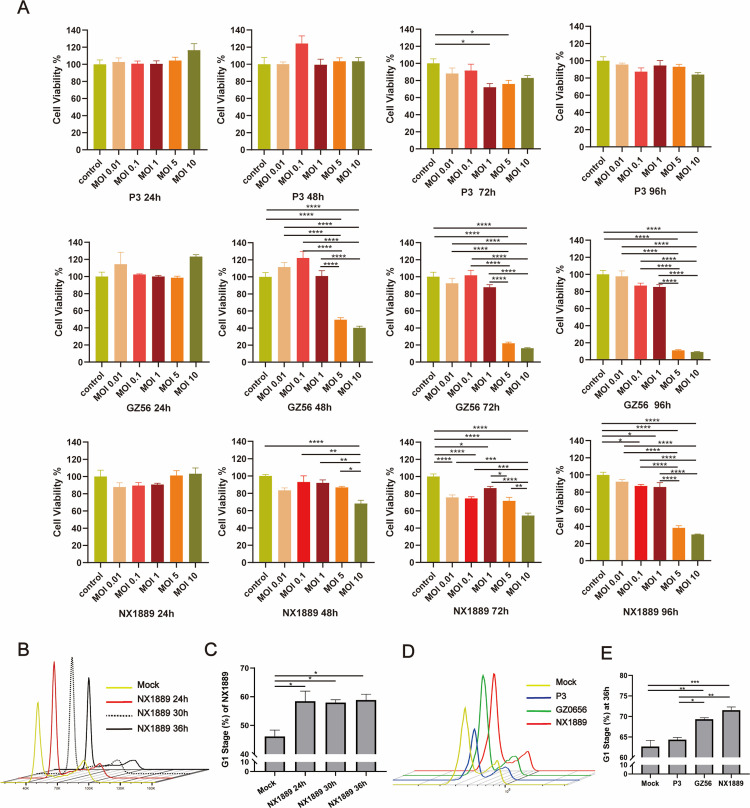
Impact of JEVs (P3, GZ56, and NX1889) infection on proliferation and cell cycle of RSC96 cells. (A) Effects of JEVs infection on the proliferation of RSC96 cells at different infection times and MOIs. Cell viability (proliferation) was quantified using Cell Counting Kit-8 (CCK-8) assay. Data normalized to uninfected controls; *n* = 5. (B, C) Impact of NX1889 infection on the cell cycle of RSC96 cells at different infection times. RSC96 cells infected with NX1889 (MOI = 1) were harvested at 24, 30, and 36 hpi, and detected by flow cytometry; *n* = 3. (D, E) Impact of JEVs infection (MOI = 1) on the cell cycle of RSC96 cells at 36 hpi; *n* = 3.

To determine whether JEV replication in SCs affects cell proliferation, we compared two GI strains that effectively replicate in SCs—NX1889 and GZ56—with GIII P3, which exhibits limited replication in SCs. Both GZ56 and NX1889 inhibited cell proliferation in a time- and dose-dependent manner, whereas P3 did not significantly influence cell proliferation (Figs 6A and S2). Prolonged infection with NX1889 inhibited cell proliferation even at low MOIs, whereas GZ56 did not (Fig 6A). Next, we investigated whether JEVs could inhibit SC proliferation by affecting cell cycle progression. It was found NX1889 infection induced G1 phase arrest in RSC96 cells at 24, 30, and 36 hpi (Fig 6B and 6C). At 36 hpi, both NX1889 and GZ56 induced G1 phase arrest; NX1889 had a more pronounced effect. In contrast, P3 did not induce G1 phase arrest (Fig 6D and 6E). These findings suggest that GI JEV inhibits SC proliferation by causing G1 phase arrest.

### 3.7 Prolonged high-dose infection with NX1889 induces apoptosis in SCs

**Fig 7:**
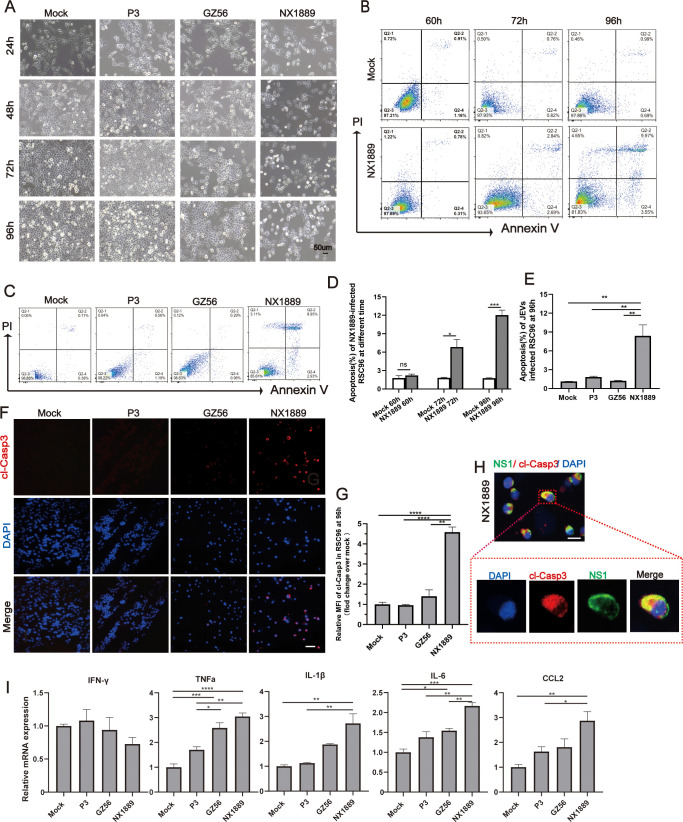
Impact of strain P3, GZ56, and NX1889 infection on apoptosis in RSC96 cells. (A) Morphology of JEVs-infected RSC96 cells at different time (MOI = 3). (B, D) Representative images and statistical analysis of apoptosis in NX1889-infected RSC96 cells at different times detected by flow cytometry (MOI = 3). (C, E) Representative images and statistical analysis of apoptosis in JEVs-infected RSC96 cells at 96 hpi detected by flow cytometry (MOI = 3). (F, G) Representative images and statistical analysis of cl-Casp3 protein of JEVs-infected RSC96 cells at 96 hpi (MOI = 3). (H) Co-localization of cl-Casp3 with JEV NS1 in NX1889-infected RSC96 cells at 96 hpi (MOI = 3). (I) Relative mRNA expression of pro-inflammatory factors in JEVs-infected RSC96 cells at 12 hpi. (MOI = 3). *n* = 3. The Scale bar is 50 μm for (A) and (F), and 20 μm for (H).

Subsequently, we investigated whether JEV infection could cause cellular damage. No typical cytopathic effects were observed in RSC96 cells infected with JEVs at MOIs of 0.01 or 0.1. However, at MOIs > 1 and with prolonged infection, large numbers of RSC96 cells in the NX1889 group exhibited hypertrophy, rounding, or detachment, whereas no typical cytopathic effects were observed in the GZ56 or P3 group (Fig 7A). Flow cytometry was utilized to detect apoptosis. When RSC96 cells were infected with NX1889 at an MOI of 3, significant apoptosis was detected starting at 72 hpi (**P* *= 0.015), coinciding with the decline in intracellular JEV load (Fig 5A). Apoptosis peaked at 96 hpi (**P* *= 0.0002, Fig 7B and 7D). At 96 hpi, among the NX1889, GZ56 and P3 groups (MOI = 3), only NX1889 induced apoptosis, as revealed by flow cytometry (Fig 7C and 7E) and fluorescence staining of cleaved caspase-3(cl-Casp3) (Fig 7F and 7G). The cl-Casp3 protein displayed co-localization with JEV NS1 protein in NX1889-infected RSC96 cells, confirming a direct correlation between viral infection and apoptosis (Fig 7H). Additionally, both GI strains (especially NX1889) elicited increased mRNA expression of several pro-inflammatory factors in RSC96 cells (Fig 7I).

### 3.8 Limb motor deficits are positively correlated with viral load and CCL2 expression in the sciatic nerves of NX1889-infected mice

**Fig 8:**
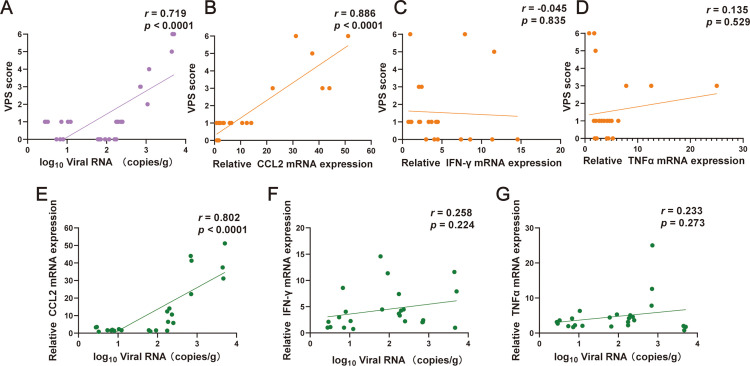
Correlation analysis among limb motor deficits, viral load, and inflammatory response of the sciatic nerves in NX1889-infected mice. Sciatic nerves were obtained between 8 to 12 dpi, and JEV RNA load and relative mRNA expression of pro-inflammatory factors were determined by qRT-PCR. (A) Correlation analysis between VPS score and JEV RNA load of the sciatic nerves; *n* = 30. (B-D) Correlation analysis between VPS score and the relative mRNA expression of (B) CCL2, (C) IFN-γ and (D) TNF-α in the sciatic nerves; *n* = 24. (E-G) Correlation analysis between JEV RNA load and the relative mRNA expression of (E) CCL2, (F) IFN-γ, and (G) TNF-α in the sciatic nerves; *n* = 24.

To further investigate the relationships among viral replication, inflammatory responses, and limb motor deficits (assessed by VPS score), we expanded the cohort of NX1889-infected mice. Correlation analysis revealed a positive correlation between VPS score and viral load in the sciatic nerves (Fig 8A). Among pro-inflammatory factors IFN-γ, TNF-a, and CCL2 in the sciatic nerves, only CCL2 levels exhibited a positive correlation with VPS scores (Fig 8B-8D). Further analysis indicated that, CCL2 levels were also positively correlated with viral loads in the sciatic nerves (Fig 8E-8G). These results suggest that both viral replication and inflammatory responses in the PNS both contribute to the development of PNI.

## 4 Discussion

Previous studies investigated the effects of different doses of the JEV NX1889 strain on peripheral nerves in mice at multiple time points post-infection, confirming that JEV NX1889 infection can induce PNI in mice [10,11]. However, several critical questions remain unresolved. It is still unknown whether the NX1889 strain could infect myelin-related cells and whether viral infection could directly cause the injury of peripheral nerves. Furthermore, the potential strain or genotype specificity of JEV-induced PNI has yet to be elucidated [10,11]. In this study, we selected four JEV strains representing the three prevalent genotypes in China: GI, GIII, and GV. GI JEV was represented by two strains: GZ56 and NX1889. GZ56 was isolated from the CSF of an infant patient with JE, whereas NX1889 was isolated from the CSF of an adult patient with JE and GBS during an outbreak. Using in vivo and in vitro model systems, we concluded that JEV strains exhibit distinct abilities to induce PNI, and the variation is likely specific to characteristic of strain NX1889 rather than a genotype-specific feature of all GI JEVs. The PNS serves as a vital replication site for JEV NX1889, in addition to its well-established CNS tropism, highlighting its broader neurotropic potential. Mechanistic investigations indicate that the cytotoxicity of JEV strain NX1889 towards SCs post infection is a vital cause of PNI, which broadens our understanding of the pathogen spectrum capable of inducing PNI via direct invasion. Additionally, the immune inflammatory response activated by SC infection results in high CCL2 expression, which attracts large numbers of CD45 + inflammatory cells to peripheral nerves, thereby exacerbating JEV-induced neurotoxicity. These findings may provide additional novel insights into the pathogenesis of GBS.

Zika virus (ZIKV) and JEV both are neurotropic flaviviruses and associated with GBS outbreak. Studies of ZIKV have led to conflicting views regarding PNI: some evidence suggests that PNI is unlikely to result from direct viral infection of the PNS [18], whereas other evidence implies that viral infection of the PNS directly causes neurological damage [14,19]. Our study showed that JEV NX1889 infection exhibited peripheral nerve tropism. Substantial viral replication persisted in the PNS, even when viral replication was minimal in peripheral organs. JEV loads in the sciatic nerves were positively correlated with the degrees of limb paralysis. In vitro, long-term, high-dose infection with NX1889 induced cytopathy in SCs. These findings suggest that viral infection of the PNS directly causes neurological damage. Volpi reported that ZIKV PRVABC59 and MR766 could infect and affect neurons and SCs in DRG explants; the two strains differed in virulence and PNS susceptibility [19]. Our study also revealed differences in PNS susceptibility and virulence among JEV strains, highlighting the specificity of strain NX1889 for the PNS.

Some RNA viruses can manipulate host cell cycles and apoptosis during infection to facilitate or influence replication. To clarify the role of direct infection in demyelination, we explored the relationship of JEV replication with SC proliferation and cell cycle, as well as the relationship of JEV replication with SC injury and apoptosis. Previous research has demonstrated differences in SC susceptibility to ZIKV, yellow fever virus 17D (YFV), and dengue virus type 2 (DENV2); it has also shown differences in SC susceptibility among ZIKV strains [20]. In the present study, we found that SCs were relatively susceptible to GI GZ56 and NX1889 but not GIII or GV strain. Although SCs were susceptible to both GI strains and SC infection with these strains could cause G1 arrest and inhibit proliferation, there were differences in the required dosage. Compared with GZ56, NX1889 could influence SC proliferation at a lower MOI. We further found that only NX1889 can induce apoptosis in these cells. This finding confirms the specificity of strain NX1889 to PNS and its corresponding neurotoxicity. Similar to our findings, some ZIKV strains reportedly can undergo direct infection and induce cell death or apoptosis in SCs and peripheral neurons [14,19]. In our study, NX1889-infected SCs began to show substantial levels of apoptosis at 72 hpi, coinciding with a significant decrease in viral titer. This decrease may be caused by direct damage to host cells, which hinders viral replication and survival. Flavivirus infection can induce endoplasmic reticulum stress, autophagy, and activation of innate immunity in host cells. These processes regulate antiviral responses and cellular survival or homeostasis, ultimately influencing viral pathogenesis [21]. Therefore, the decrease in viral titer may also be attributed to the activation of host cell antiviral responses triggered by extensive viral replication.

In addition to virus-induced cytotoxicity, immunoinflammatory responses contribute to the development of JEV-induced PNI. NX1889 infection upregulated the expression levels of various inflammatory factors both in vivo and in vitro. Furthermore, CCL2 levels in the sciatic nerves were positively correlated with both viral load of the sciatic nerves and VPS score, suggesting that it plays a crucial role in the pathogenesis of PNI. In the PNS, CCL2 is primarily secreted by SCs and is known to attract migrating monocytes [22] and macrophages [23] to the PNS; this process is closely associated with neuralgia, motor deficits, and nerve repair and regeneration [24,25]. Based on these, NX1889-infected SCs increased CCL2 expression, which can lead to the recruitment of inflammatory cells to the PNS, and further exacerbate JEV-induced neurotoxicity.

GBS is an acute post-infectious inflammatory immune-mediated peripheral neuropathy. Although most frequently linked to *Campylobacter jejuni*, other agents (e.g., *Mycoplasma pneumoniae*; cytomegalovirus; Epstein–Barr virus; hepatitis A, B, and E viruses; ZIKV; DENV; West Nile virus; alphavirus, and severe acute respiratory syndrome coronavirus 2 [SARS-CoV-2]) are potentially associated with GBS [12,26,27]. The complex immune and inflammatory responses triggered by pathogen infections play key roles in GBS pathogenesis. *C. jejuni*-mediated molecular mimicry is a widely recognized mechanism for GBS development [28]. Hepatitis B virus-mediated vascular injury, a possible GBS-inducing mechanism, involves immune complex deposition in the peri-neural vascular endothelium, leading to secondary vascular inflammation and nerve ischemia; this process potentially results in GBS [29]. Another study found that human SC supported alphavirus entry and replication, and alphaviral infection of SC engaged a canonical innate immune response, but failed to affect SC regenerative properties [30]. Reports of nerve injury by direct PNS invasion in GBS are limited. Our study revealed that JEV is capable of replicating within SCs and impairing their viability, and productive viral replication over a long period of time even leads to cell death, which directly results in peripheral demyelination. Additionally, the subsequent immunoinflammatory response post SC infection exacerbates damage. These findings may offer additional novel perspectives on the pathogenesis of GBS.

Through comparative sequence analysis, we identified distinct variations in the amino acid sequences and glycosylation sites within the E protein between the GI NX1889 strain and three other strains (GI GZ56, GIII P3, and GV XZ0934) (S3 Fig). These differences may account for the observed variations in pathogenic potential among these strains, but further validation through reverse genetics approaches is still required. Furthermore, due to the neuroanatomical and functional divergence between murine models and human peripheral nervous system, findings derived from mouse models cannot be directly extrapolated to human pathophysiology. The existence of an analogous pathogenesis in humans remains to be further substantiated.

In conclusion, our research demonstrates that JEVs exhibit distinct ability to induce PNI; the differences prone to be strain-specific, rather than genotype-specific. A previous study showed that patients with JE and PNI experience more severe symptoms and worse prognoses relative to patients with JE alone. The medical community must remain vigilant about the potential public health and safety threat posed by the increasing spread of strain NX1889 and related variants. A better understanding of the mechanisms by which JEV induces PNI may facilitate the development of preventive and therapeutic strategies to mitigate nerve injury. Our results support a previously unrecognized mechanism: peripheral nerves or SCs are susceptible to strain NX1889 and its toxicity, both in vivo and in vitro, and the subsequent inflammatory damage is also linked to viral replication. These findings indicate that antiviral treatment may be a crucial component of nerve injury mitigation, providing insights into developing treatment strategies for JEV-associated GBS.

## Supporting information

S1 Fig Plaque morphology of four JEV strains. The plaque was formed in BHK21 cells.
(TIF)

S2 Fig Impact of NX1889 infection at varying MOIs on RSC96 cell proliferation. RSC96 cells were uniformly seeded and infected with JEV NX1889 strain at indicated MOIs. Representative photomicrographs showed cellular density at 72 hpi.
(TIF)

S3 Fig Comparative analysis of the amino acid sequences and glycosylation sites of the E protein of JEV strains. The sequence alignment was performed using the NetOGlyc 4.0 server (available at https://services.healthtech.dtu.dk/services/NetOGlyc-4.0). Glycosylation sites are highlighted in red.
(TIF)

S1 Table The background of JEV strains. (DOCX)

S2 Table Viral paralysis scale. (DOCX)

S3 Table List of primers used in the study. (DOCX)

S4 Table Electrophysiological results of the bilateral sciatic nerves in C57BL/6 mice inoculated with JEVs. (DOCX)

S1 Data The peripheral nerve injury mediated by JEV strain NX1889 infection and impairment of Schwann cells. (ZIP)
