# Supplementary figures and images for: Peripheral nerve injury mediated by JEV strain NX1889 infection and impairment of Schwann cells
Source: PLoS Negl Trop Dis. 2025 Aug 26;19(8):e0013466. doi: 10.1371/journal.pntd.0013466 (PMC12410878; doi:10.1371/journal.pntd.0013466)

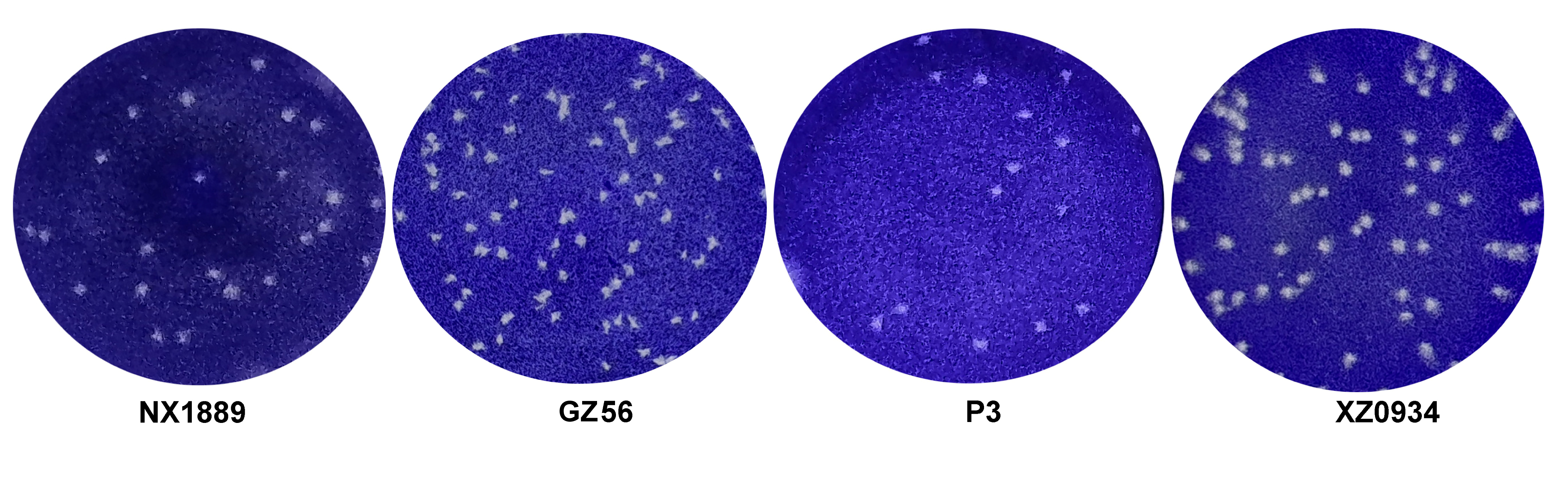

Supplement: S1 Fig — The plaque was formed in BHK21 cells. (TIF) [file pntd.0013466.s001.tif]

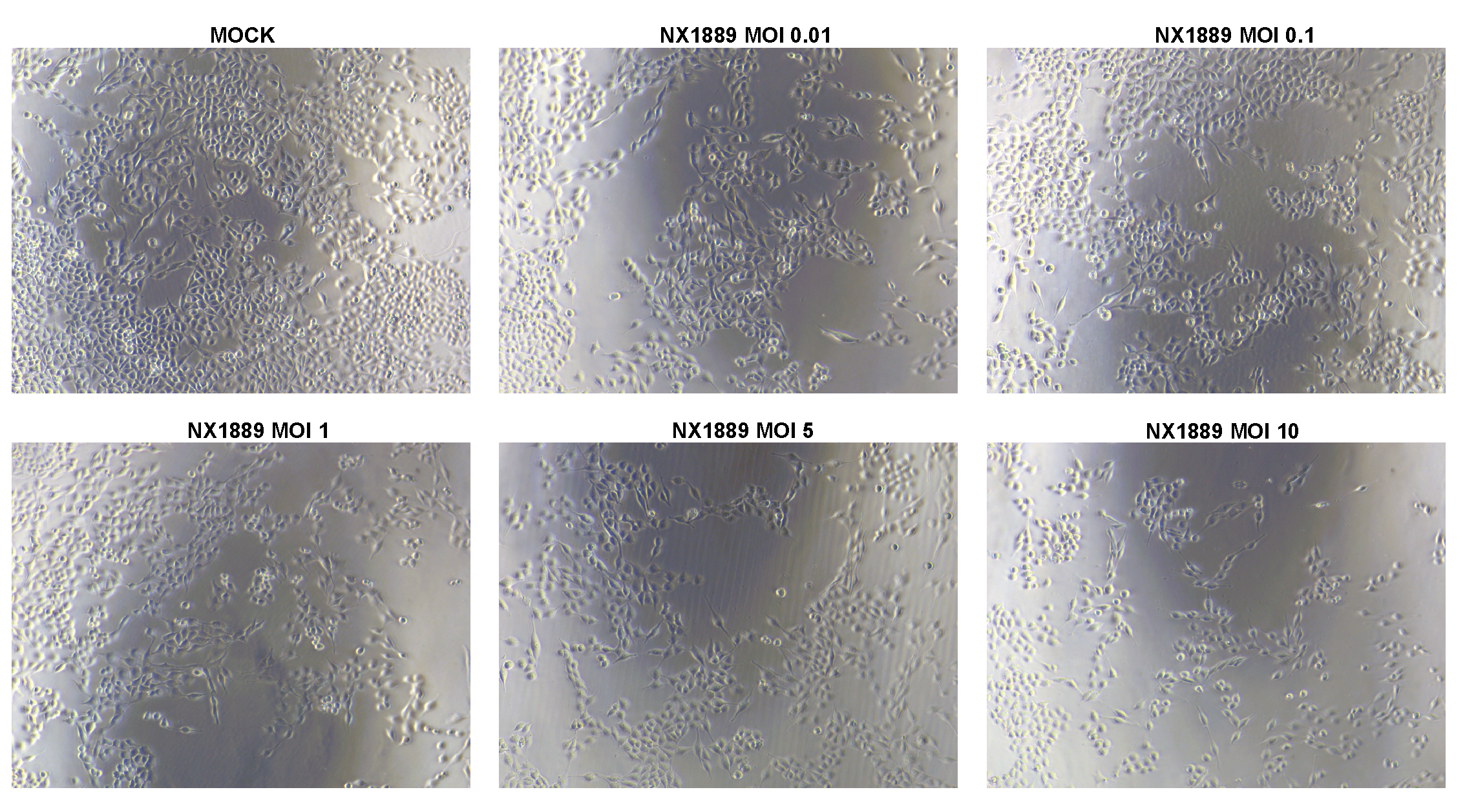

Supplement: S2 Fig — RSC96 cells were uniformly seeded and infected with JEV NX1889 strain at indicated MOIs. Representative photomicrographs showed cellular density at 72 hpi. (TIF) [file pntd.0013466.s002.tif]

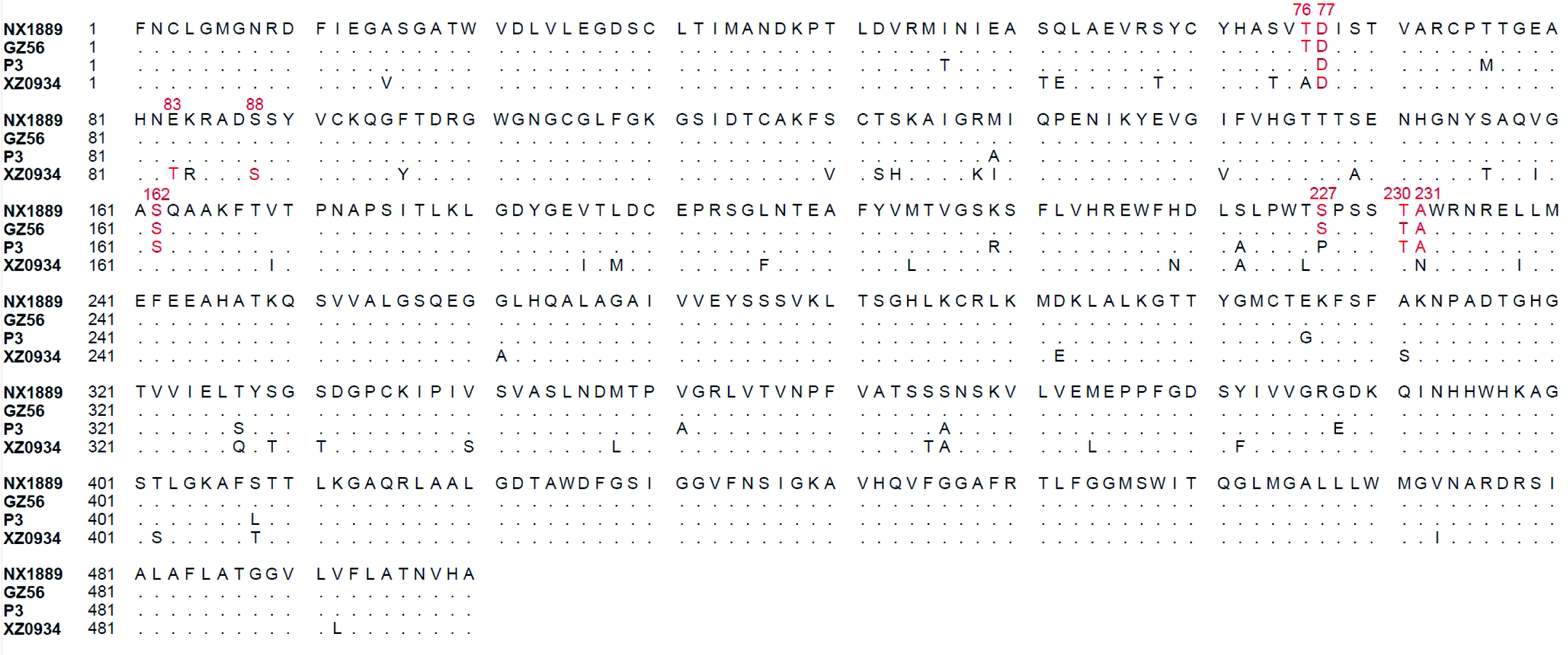

Supplement: S3 Fig — The sequence alignment was performed using the NetOGlyc 4.0 server (available at https://services.healthtech.dtu.dk/services/NetOGlyc-4.0). Glycosylation sites are highlighted in red. (TIF) [file pntd.0013466.s003.tif]
