# Supplementary material for: Peripheral nerve injury mediated by JEV strain NX1889 infection and impairment of Schwann cells
Source: PLoS Negl Trop Dis. 2025 Aug 26;19(8):e0013466. doi: 10.1371/journal.pntd.0013466 (PMC12410878; doi:10.1371/journal.pntd.0013466)
Supplement: S1 Table — (DOCX) [file pntd.0013466.s004.docx]

**S1Table**. The background of JEV strains.

| **Strain** | **Year** | **Site** | **Host** | **Age (y)** | **Gender** | **Genotype** | **GenBank No.** |
| --- | --- | --- | --- | --- | --- | --- | --- |
| NX1889 | 2018 | Ningxia | CSF of human | 53 | Male | GIb | MT134112 |
| GZ56 | 2008 | Guizhou | CSF of human | 0.5 | Female | GIb | HM366552 |
| P3 | 1950 | Beijing | Brain of human | 15 | Female | GIII | U47032 |
| XZ0934 | 2009 | Tibet | Mosquito | - | - | GV | JF915894 |

Abbreviations. CSF: cerebrospinal fluid, y: years.
