# Supplementary material for: Peripheral nerve injury mediated by JEV strain NX1889 infection and impairment of Schwann cells
Source: PLoS Negl Trop Dis. 2025 Aug 26;19(8):e0013466. doi: 10.1371/journal.pntd.0013466 (PMC12410878; doi:10.1371/journal.pntd.0013466)
Supplement: S2 Table — (DOCX) [file pntd.0013466.s005.docx]

**S2 Table.** Viral paralysis scale.

| **Score** | **Description** | **Signs** |
| --- | --- | --- |
| 0  1  2  3  4  5  6 | normal  onset of symptoms  mild paresis  moderate paresi  severe paresis  paralysis  complete paralysis or death | weight-bearing, plantar stepping with tail up during walking  weight-bearing, tail position may be down or not fully up during walking, plantar stepping with mild rotation, wobble  limp, tail down during walking, mild miss-steps, stiffer joint movement  obvious weakness of limb (but able to bear weight), moderate miss-steps, stiffer joint movement  not bearing much weight, severe miss-steps, obviously decreased joint movement  no weight-bearing steps, slight joint movement  no weight bearing steps, no joint movement |
