# Supplementary material for: Peripheral nerve injury mediated by JEV strain NX1889 infection and impairment of Schwann cells
Source: PLoS Negl Trop Dis. 2025 Aug 26;19(8):e0013466. doi: 10.1371/journal.pntd.0013466 (PMC12410878; doi:10.1371/journal.pntd.0013466)
Supplement: S3 Table — (DOCX) [file pntd.0013466.s006.docx]

**S3 Table**. List of primers used in the study.

| **Target** | **Primer Sequences (5’-3’)** | |
| --- | --- | --- |
| TNF-α(Rat) | F | GCGTGTTCATCCGTTCTCTACC |
| IFN-γ (Rat)  IL-6(Rat)  IL-1β (Rat)  CCL2 (Rat)  GAPDH (Rat)  TNF-α (Mouse)  IFN-γ(Mouse)  IL-6(Mouse)  IL-1β(Mouse)  CCL2(Mouse)  GAPDH(Mouse) | R  F  R  F  R  F  R  F  R  F  R  F  R  F  R  F  R  F  R  F  R  F  R | TACTTCAGCGTCTCGTGTGTTTCT  CAACCCACAGATCCAGCACAAAG  TCCGCTTCCTTAGGCTAGATTCTG  AGTTGCCTTCTTGGGACTGATGT  GGTCTGTTGTGGGTGGTATCCTC  TACATCAGCACCTCTCAAGC  GTCAACTATGTCCCGACCA  ACCACTATGCAGGTCTCTGTCA  GGCATTAACTGCATCTGGCTGA  GACATGCCGCCTGGAGAAAC  AGCCCAGGATGCCCTTTAGT  CATCTTCTCAAAATTCGAGTGACAA  TGGGAGTAGACAAGGTACAACCC  AGCGGCTGACTGAACTCAGATTGTAG  GTCACAGTTTTCAGCTGTATAGGG  AGCCAGAGTCCTTCAGA  GGTCCTTAGCCACTCCT  GCCTTGGGCCTCAAAGGAAAGAATC  GGAAGACACGGATTCCATGGTGAAG  ACTGAAGCCAGCTCTCTCTTCCTC  TTCCTTCTTGGGGTCAGCACAGAC  GGTGAAGGTCGGTGTGAACG  CTCGCTCCTGGAAGATGGTG |

Abbreviations. F: forward, R: reverse.
