# Supplementary material for: Peripheral nerve injury mediated by JEV strain NX1889 infection and impairment of Schwann cells
Source: PLoS Negl Trop Dis. 2025 Aug 26;19(8):e0013466. doi: 10.1371/journal.pntd.0013466 (PMC12410878; doi:10.1371/journal.pntd.0013466)
Supplement: S4 Table — (DOCX) [file pntd.0013466.s007.docx]

**S4 Table**. Electrophysiological results of the bilateral sciatic nerves in C57BL/6 mice inoculated with JEVs.

| **Number** | **side** | **NCV**  **(m/s）** | **Ampli**  **(mV)** | **End**  **Latency(ms)** | **side** | **NCV**  **(m/s）** | **Ampli**  **(mV)** | **End**  **latency(ms)** |
| --- | --- | --- | --- | --- | --- | --- | --- | --- |
| Mock-1 | L | 50.00 | 10.30 | 1.28 | R | 50.00 | 21.00 | 1.32 |
| Mock-2 | L | 50.00 | 20.20 | 0.84 | R | 50.00 | 20.60 | 1.04 |
| Mock-3 | L | 50.00 | 20.60 | 1.04 | R | 58.30 | 17.10 | 0.96 |
| Mock-4 | L | 50.00 | 22.10 | 1.16 | R | 43.80 | 12.50 | 1.16 |
| Mock-5 | L | 37.50 | 10.40 | 1.00 | R | 43.80 | 14.70 | 1.16 |
| Mock-6 | L | 50.00 | 10.90 | 1.32 | R | 43.80 | 11.90 | 1.36 |
| NX1889-1 | L | 10.40 | 12.10 | 1.00 | R | 15.60 | 13.10 | 1.28 |
| NX1889-2 | L | 18.80 | 6.74 | 2.20 | R | 8.93 | 7.86 | 1.04 |
| NX1889-3 | L | 31.00 | 6.46 | 1.60 | R | 18.70 | 4.85 | 1.64 |
| NX1889-4 | L | 9.37 | 5.84 | 1.84 | R | 11.40 | 7.12 | 1.36 |
| NX1889-5 | L | 15.60 | 7.62 | 1.64 | R | 15.60 | 12.40 | 1.20 |
| NX1889-6 | L | 13.90 | 8.29 | 2.56 | R | 10.40 | 9.20 | 1.60 |
| GZ56-1 | L | 25.00 | 11.10 | 1.12 | R | 37.50 | 9.42 | 1.96 |
| GZ56-2 | L | 35.00 | 13.20 | 1.00 | R | 21.90 | 13.80 | 1.12 |
| GZ56-3 | L | 30.00 | 21.90 | 1.36 | R | 43.70 | 19.50 | 1.04 |
| GZ56-4 | L | 43.80 | 12.90 | 0.88 | R | 43.80 | 15.40 | 1.88 |
| GZ56-5 | L | 58.30 | 12.70 | 1.08 | R | 43.70 | 13.20 | 1.04 |
| GZ56-6 | L | 37.50 | 9.32 | 1.64 | R | 37.50 | 14.40 | 1.80 |
| P3-1 | L | 50.00 | 14.30 | 1.04 | R | 43.80 | 19.60 | 1.40 |
| P3-2 | L | 43.70 | 15.80 | 1.60 | R | 43.80 | 11.80 | 1.76 |
| P3-3 | L | 50.00 | 18.60 | 1.36 | R | 43.80 | 12.60 | 1.52 |
| P3-4 | L | 50.00 | 16.60 | 1.00 | R | 58.30 | 11.50 | 1.60 |
| P3-5 | L | 58.30 | 11.50 | 1.44 | R | 58.30 | 14.10 | 1.48 |
| P3-6 | L | 50.00 | 13.30 | 1.08 | R | 43.80 | 12.40 | 1.48 |
| XZ0934-1 | L | 30.00 | 16.20 | 1.24 | R | 25.00 | 22.20 | 1.24 |
| XZ0934-2 | L | 30.00 | 14.70 | 1.44 | R | 37.50 | 10.50 | 1.04 |
| XZ0934-3 | L | 37.50 | 9.57 | 1.48 | R | 21.40 | 11.60 | 1.60 |
| XZ0934-4 | L | 30.00 | 12.90 | 1.64 | R | 37.30 | 10.20 | 1.32 |

Abbreviations. L: left, R: right, NCV: nerve conduction velocity, Ampli: amplitude of the distal compound muscle action potential.
